# Individual and household risk factors of severe acute malnutrition among under-five children in Mao, Chad: a matched case-control study

**DOI:** 10.1186/s13690-018-0281-5

**Published:** 2018-08-01

**Authors:** Jovana Dodos, Chiara Altare, Mahamat Bechir, Mark Myatt, Brigitte Pedro, Francois Bellet, Jean Lapegue, Joachim Peeters, Mathias Altmann

**Affiliations:** 1 Action Contre la Faim, 14/16 Boulevard Douaumont - CS 80060, 75854 PARIS, CEDEX 17 France; 2 Alliance Sahélienne de Recherches Appliquées pour le Développement Durable ASRADD, N’djamena, Chad; 3 Brixton Health, Powys, UK; 4 UNICEF Chad, N’djamena, Chad; 5 UNICEF Regional Office for West and Central Africa, Dakar, Senegal

**Keywords:** Severe acute malnutrition, Childhood illnesses, Household determinants, Risk factors

## Abstract

**Background:**

Severe acute malnutrition (SAM) is one of the leading causes of morbidity and mortality in Chad. The reasons behind persistently high prevalence of SAM in the Kanem region are still poorly understood, leaving national and international partners without clearly identified drivers to address. Current knowledge of SAM determinants in this context is largely based on very limited data. The aim of this study was thus to investigate individual and household-level risk factors for SAM among under-five children in Mao health district.

**Methods:**

A matched case-control study was conducted on 411 (137 cases and 274 controls) children aged 6–59 months with their caretakers from mid-February to August 2017. Data were collected by using a structured interviewer administered questionnaire, anthropometric measurements and through direct observations of household environment. Controls were matched to their cases on place of residence and on age (± 3 months). Data were double-entered, processed and analysed using Epi Info 7.2.0.1. Conditional logistic regression was used to analyse the association of independent variables with SAM. For multivariable analysis, two models were constructed to investigate risk factors for SAM, at individual and household level. A stepwise backwards elimination approach with a significance level of *p* = 0.05 was used to build the final models.

**Results:**

At the individual level, SAM was significantly associated with diarrhoea [AOR (95% CI) = 10.7 (4.2–27.3)], fever [AOR (95% CI) = 8.4 (3.1–22.8)], vomiting [AOR (95% CI) = 7.6 (3.0–19.7)], being stunted [AOR (95% CI) = 5.3 (1.7–16.3)], and type of complementary meal [AOR (95% CI) = 4.4 (1.0–19.6)]. At the household level, SAM was significantly associated with undernourished caretaker [AOR (95% CI) = 2.6 (1.2–5.5)], caretaker’s hand washing habits [AOR (95% CI) = 1.9 (1.2–3.1)], absence of toilet [AOR (95% CI) = 1.9 (1.1–3.6)], caretaker’s marriage status [AOR (95% CI) = 7.7 (2.0–30.1)], and low household food diversity [AOR (95% CI) = 1.8 (1.0–3.1)].

**Conclusion:**

The present study identified the need to address both treatment and prevention of infections in children through an integrated approach. Well-organized efforts to improve child feeding practices, household hygiene and sanitation conditions, women’s nutritional status, along with increasing household food diversity are likely to lead to improved nutritional status of children in this setting.

## Background

Severe acute malnutrition (SAM) is the most extreme form of acute undernutrition, resulting from acute food shortages, a recent bout of illness, inappropriate child care or feeding practices, or a combination of these factors. SAM is defined by a low mid-upper-arm-circumference (MUAC), and/or a weight-for-height below − 3 Z-scores of the median WHO growth standards [1] and/or presence of bilateral pitting oedema [2].

Severely malnourished children have weakened immunity, are susceptible to long term developmental delays, and have 5–20 times higher risk of death compared to well-nourished children [3]. SAM can be a direct cause of child death, or it can act as an indirect cause by dramatically increasing the case fatality rate in children suffering from common childhood illnesses such as diarrhoea, pneumonia or measles. Children experiencing SAM can face long-term consequences for their future health, learning and economic performance [4].

In 2016, nearly 52 million children under-five suffered from acute malnutrition and 17 million from severe acute malnutrition [5]. Southern Asia and Sub-Saharan Africa bear the greatest share of SAM children; around 75% of all severely malnourished children live in lower-middle income countries [5], including Chad, where the prevalence of SAM is persistently high.

Chad, after Somalia, has the second highest under-five mortality rate in the world (139 deaths per 1000 live births in 2015) [6, 7]. In order to address the critical nutrition situation in the country, the Ministry of Health of the Republic of Chad has been implementing programmes to strengthen nutritional surveillance and overall management of acute malnutrition. Despite all the efforts made by the Government and its technical and financial partners, the results of 2017 nutrition survey are concerning: prevalence of SAM was estimated at 3.9% and of Global Acute Malnutrition (GAM) at 13.9%. Nutritional situation in the country deteriorated compared to 2016, when the prevalence of SAM and GAM at the national level was 2.6 and 11.9%, respectively. The Sahelian zone of the country has been especially affected. GAM prevalence among children under five in the Kanem region was estimated at 19.2% [15.4–23.7], well beyond the commonly used threshold for a public health emergency [8].

Action Contre la Faim (ACF), an international non-governmental organization, has been working in Kanem since 1980s. According to a contextual nutrition causal analysis conducted in Kanem in 2012 [9], major drivers of undernutrition include high prevalence of childhood illnesses, poor access to healthcare, climatic shocks, insufficient agricultural production, poor access to safe drinking water, inadequate hygiene practices, insufficient access to markets and high maternal workload. Building upon this evidence, we strived to identify and quantify individual and household-level risk factors leading to SAM among under-five children in order to achieve greater understanding of SAM determinants and assess the strength of association. This would allow more effective design and prioritisation of interventions, in addition to better targeting of the most vulnerable populations.

## Methods

### Study setting and design

ACF and its partner in Chad Alliance Sahélienne de Recherches Appliquées pour le Développement Durable (ASRADD), conducted a matched case-control study in Mao city, the capital of Kanem region, Chad. Set in a desert area, Mao city is located in a remote zone, difficult to reach. It has an estimated population of 19,000 inhabitants [10]. The catchment area of Mao health centre covers 54 villages within a 10 km radius.

### Selection of cases

Cases were defined as SAM children, between six and 59 months of age, newly admitted, relapsed or readmitted to the nutritional Outpatient Therapeutic Program (OTP) of Mao health centre. Anthropometric criteria for admission to the OTP were as follows: MUAC < 115 mm and/or weight for height Z-score (WHZ) < − 3 standard deviations (SD) and/or presence of bilateral oedema. Children who did not reside in the 54 villages under the jurisdiction of Mao health centre and those who we were not able to follow up during household visits (due to incorrect data provided by caretakers at the time of initial interview in the health centre) were not eligible for inclusion in the study.

### Selection of controls

Controls were children aged six to 59 months, not undernourished (defined as MUAC ≥125 mm and weight for height Z-score (WHZ) of ≥ − 1.5 SD on the day of the survey and without signs of bilateral oedema), in good general health and without known past episodes of SAM.

In order to study specifically individual and household-level risk factors, controls were matched to their cases on their place of residence (village / neighbourhood). We also matched cases and controls on age (± 3 months), as it is a potential cofounder of the association.

### Sample size calculation

Sample size was calculated using standard formula for a matched case-control study [11]. As several risk factors were considered, a proportion of 80% among controls, e.g. proportion of households without tap water [12], was selected in order to yield the maximum sample size. We used power (1 – β) of 80% and significance (α) of 5%; control to case ratio of 2:1 to detect an odds ratio of 3 (considered as a moderately strong risk factor). With the given parameters, the sample size required for the study was 360, collected as 120 cases and 240 controls. Resources allowed to increase the sample size to 411, including 137 cases and 274 controls. Such an increment in sample size augmented the power of the study.

### Data collection instruments and procedure

Data collection lasted from the beginning of February to mid-August 2017.

Cases were recruited during the admission days to the OTP in Mao health centre. Anthropometric measures were extracted from the OTP register. Data on health and nutritional status of caretaker, household WASH (water, sanitation and hygiene) conditions, socio-economic and demographic data were collected on the following day during the household visit through face-to-face interview, using a structured interviewer administered questionnaire, and direct observations.

Controls were recruited from the same neighbourhood where cases reside. After completing the interview with the case caretaker, data collectors visited the closest neighbouring households to identify eligible children for the controls. Anthropometric measurements of the controls were conducted at their household by the two team members in charge of this task. Weight (kg, g) was measured using UNICEF electronic scale (to the nearest of 0.1 kg); length and height (cm) were measured by UNICEF portable measuring board (to the nearest mm). Presence of oedema was identified if a bilateral depression remained 3 sec after the pressure was released.

The questionnaire was based on the review of available literature, findings from two nutrition causal analyses studies (“Link NCA”) conducted in Kanem [9] and Ouaddaï region [13], as well as internal consultations with ACF’s program managers in Mao.

The questionnaire was developed in French and translated into local languages (*Goran and Kanembou*) through a group discussion with data collectors who had a good command of both French and the local languages. Data collectors were recruited locally from a pool of enumerators who had previously worked with ACF and ASRADD. They were trained for 2 days by the principal investigator in filling out the questionnaire and use of measurement devices. Each data collector was accompanied by the principal investigator for at least 3 interviews during the survey pilot, to provide hands-on training. Data collectors were divided in two teams, each with a supervisor who ensured the quality control of the collected data.

### Variables included in the study

For the purpose of this study, we broadly categorized risk factors for SAM into two groups: individual and household-level risk factors for SAM.

### Individual risk factors

Individual risk factors included sex, diet, recent morbidity, stunting and child care and feeding practices. Age of cases was copied from the OTP registers. Age of controls was identified by asking caretakers about the age of a child. To identify retrospective morbidity in children, caretakers were asked about any occurrence of disease during the past 15 days. Diarrhoea was defined as three or more loose or liquid stools per day. Stunting in children was defined as a height-for-age Z-score (HAZ) < − 2 SD as per 2006 WHO Growth Standards. Immunization status of the children was assessed based on vaccination card, if present. Signs of traditional healing practices were identified by asking caretakers if child experienced recent dental extraction, removal of uvula, scarification and/or burns. Early complementary feeding was defined as introducing foods or liquids other than breast milk before or at 6 months of age.

### Household-level risk factors

Household-level risk factors included variables related to caretaker’s nutritional status, caretaker’s health seeking behaviour and personal habits, household WASH conditions, household demographic and socio-economic profile. Nutritional status of the caretakers was defined with MUAC; a threshold of 220 mm [14] was used to define undernutrition. Early pregnancy was defined as a pregnancy in women under the age of 18. Antenatal care follow-up was defined as consulting with health professionals during the last pregnancy. Recent morbidity in the household was defined as presence of symptoms (fever, diarrhoea, vomiting, cough) during the past 15 days in family members other than a child.

Samples of drinking water used for the child included in the study were taken in each household. Physio-chemical and bacteriological qualities were analysed with Wagtech® water safety kit. Good water *quality* was defined according to national standards: *Escherichia coli* per 100 ml = 0 and 0.2 < free residual chlorine < 0.5 mg/l and NTU (Nephelometric Turbidity Unit) < 5. The concentration of *Escherichia coli* per 100 ml was also used as a continuous variable in dose-response analysis. Minimum water *quantity* was defined according to the SPHERE standards [15] with a threshold set at 15 l per person per day. Hand washing knowledge was identified by reporting five key times for hand washing: before preparing food, before eating, before breastfeeding or feeding a child, after defecation/using toilet, after managing child’s faeces. Hand washing practice was identified by asking caretakers to demonstrate the way they usually wash hands (e.g. using water, using soap, rubbing hands at least three times). A hand washing behaviour score (ranging from 0 to 10) was created by summing up responses related to hand washing knowledge and hand washing practice. The ability to name five key times for hand washing were also used as independent variables with values yes/no. Environmental hygiene (e.g. presence of faecal material in the kitchen/ child’s play areas, presence of stagnant water and use of mosquito bed nets) was assessed through direct observation of the household environment.

Household Food Diversity Score (0–12) was assessed over the 7 days preceding the interview and included 12 food groups consumed at the household level. Household food diversity was defined as low if the score was below or equal to six [16]. Preoccupation of caretaker that the household will not have enough food to satisfy basic family needs was used as a proxy for food insecurity. Low income was defined as having monthly financial resources below 35.000 FCFA (Central African Financial Cooperation Franc), which corresponded to the median in our study.

### Data quality control, processing and analysis

Data collectors were paired and supervised during data collection. Before data entry, each questionnaire was checked by the supervisors for completeness and logical consistency. Overall data collection and entry were supervised by the principal investigator. Data were double-entered, processed and analysed using Epi Info 7.2.0.1.

Descriptive statistics were expressed as percentages and frequency. Conditional logistic regression for matched data was used to identify factors independently associated with the outcome. Variables that were significant in bivariate analysis were considered for inclusion in the multivariable conditional logistic regression models. A *p*-value =0.05 was considered to be significant.

For studying the risk factors of SAM at the two levels, individual and household, we treated the data as two separate matched case-control studies and constructed two models:

1. First model included risk factors at child/ individual level: sex, diet, recent morbidity, stunting, child care and feeding practices.
2. The second model included risk factors at household level: caretaker’s nutritional status, caretaker’s health seeking behaviour and personal habits, household WASH conditions, household demographic and socio-economic profile.

A stepwise backwards elimination approach with a significance level of *p* = 0.05 was used to build the final models. Multicollinearity was assessed using variance inflation factor (VIF). None of the variables had VIF > 4.

## Results

A total of 137 cases and 274 controls were included in the study. A selection of descriptive results is presented in Table 1.

**Table 1:** General characteristics of the study participants, Mao health district, February–August 2017

Characteristic	Cases *N* = 137	Controls *N* = 274	Total *N* = 411
Demographic and socio-economic profile			
Number of people in the household, mean	5.61	5.91	5.81
Number of children under five, mean	1.74	1.86	1.82
Household possesses livestock	76 (55.47%)	172 (62.77%)	248 (60.34%)
Agricultural activities by the household	32 (23.36%)	59 (21.53%)	91 (22.14%)
Child caretakers’ profile and health seeking behaviour			
Age of caretakers (years), mean	25.42	25.74	25.63
Income generating status / Not employed	125 (91.24%)	247 (90.15%)	371 (90.51%)
Follow antenatal care visits	118 (86.13%)	250 (91.24%)	368 (89.54%)
Usual practice when child is sick			
Taking child to the health centre	50 (36.50%)	117 (42.70%)	167 (40.63%)
Self-treatment methods	85 (62.04%)	156 (56.93%)	241 (58.64%)
Caretaker participates in decision making on expenses for child health care	10 (7.30%)	11 (4.01%)	21 (5.11%)
Reasons for not going to health centre to consult with health professionals			
High prices	64 (46.72%)	120 (43.80%)	184 (44.77%)
Lack of time	21 (15.33%)	43 (15.69%)	64 (15.57%)
Lack of means of transport	9 (6.57%)	28 (10.22%)	37 (9.00%)
Long distances	7 (5.11%)	13 (4.74%)	20 (4.87%)
Household WASH conditions			
Soap is present in the household	128 (93.43%)	259 (94.53%)	387 (94.16%)
Where toilet is not present, family practice open defecation	71 (51.82%)	120 (43.79%)	191 (46.47%)
Principal water source for the household			
Tap	57 (41.61%)	118 (43.07%)	175 (42.58%)
Forage with a hand pump	47 (34.31%)	89 (32.48%)	136 (33.09%)
Open well	26 (18.98%)	42 (15.33%)	68 (16.15%)
Other	7 (5.11%)	25 (9.12%)	32 (7.79%)
Time needed to collect water ≥30 min	71 (51.82%)	184 (67.15%)	255 (62.04%)
Persons in charge of water collection			
Women	132 (96.35%)	265 (96.72%)	397 (96.59%)
Men	15 (10.95%)	28 (10.22%)	43 (10.46%)
Children	75 (54.74%)	134 (48.91%)	209 (50.85%)
Household has less than 15 l per person per day	10 (7.30%)	14 (5.11%)	24 (5.84%)
Container used for water transport is dirty	70 (51.09%)	107 (39.05%)	177 (43.07%)
Water is stored in an unhygienic place	115 (83.94%)	200 (72.99%)	315 (76.64%)
Water is treated before consumption	5 (3.65%)	10 (3.65%)	15 (3.65%)
Good drinking water quality at point-of-use	0 (0.0%)	0 (0.0%)	0 (0.0%)
*Escherichia coli* = 0	28 (20.44%)	44 (16.06%)	72 (17.52%)
Presence of faecal material (human or animal) around the household	99 (72.26%)	200 (72.99%)	299 (72.57%)
Presence of faecal material (human or animal) in the child’s play areas	65 (47.45%)	124 (45.26%)	189 (45.99%)
Kitchen utensils or food leftovers are left uncovered on the floor	55 (40.15%)	96 (35.04%)	151 (36.74%)
Household uses mosquito bed nets	21 (15.33%)	35 (12.77%)	56 (13.63%)

*Cases = children 6–59 months, MUAC < 115 mm and/or weight for height Z-score (WHZ) < − 3 SD and/or presence of bilateral oedema*

*Controls = children 6–59 months, MUAC ≥125 mm and weight for height Z-score (WHZ) of ≥ − 1.5 SD, without sign of bilateral oedema*

### Individual risk factors of SAM

Average age was 15.4 and 15.8 months among cases and controls, respectively. Overall, 56% of the participants were female. For 83% of the study participants, vaccination card was not present in the household during the survey. Among those who were immunized (*N* = 70), completed immunization status according to the national program was present in 8 and 6% of cases and controls, respectively. Less than 20% of study participants were exclusively breastfed during first 6 month of life. There was no difference between cases and controls with regard to frequency of feeding.

Bivariate analyses showed that sex, recent morbidity in a child, being stunted, non-exclusive breastfeeding during first 6 months of life, type of complementary meal introduced (family dish), age when the first complementary meal was introduced (≤ 6 months), and signs of traditional healing practices on a child were significantly associated with SAM (Table 2). In the multivariable analysis, presence of diarrhoea [AOR (95% CI) = 10.7 (4.2–27.3)], fever [AOR (95% CI) = 8.4 (3.1–22.8)], vomiting [AOR (95% CI) = 7.6 (3.0–19.7)], being stunted [AOR (95% CI) = 5.3 (1.7–16.3)], and type of complementary meal introduced (family dish) [AOR (95% CI) = 4.4 (1.0–19.6)] remained significantly associated with SAM (Table 2).

**Table 2:** Individual risk factors of SAM, Mao health district, February–August 2017

Child characteristic	Cases (%)	Controls (%)	COR (95%CI)	AOR (95%CI)	*P*-value
Sex, female	80 (58.4)	150 (54.7)	4.9 (1.0–24.0)		
Diarrhoea in the last 15 days	124 (90.5)	86 (31.4)	18.9 (19.2–39.1)	10.7 (4.2–27.3)	0.000
Fever in the last 15 days	123 (89.8)	104 (38.0)	18.3 (8.5–39.8)	8.4 (3.1–22.8)	0.000
Cough in the last 15 days	87 (56.9)	67 (24.5)	4.1 (2.6–6.5)		
Vomiting in the last 15 days	75 (54.7)	27 (9.0)	12.6 (6.5–24.6)	7.6 (3.0–19.7)	0.000
Being stunted	123 (89.8)	175 (63.9)	6.77 (3.3–13.8)	5.3 (1.7–16.3)	0.004
Non-exclusive breastfeeding	120 (87.6)	221 (80.7)	1.9 (1.0–3.6)		
Complementary feeding at ≤6 months	91 (66.4)	155 (56.6)	1.6 (1.0–2.5)		
Type of first complementary meal, family dish	13 (9.5)	14 (5.1)	2. 1 (0.9–4.9)	4.4 (1.0–19.6)	0.050
Signs of traditional curing practices on a child	60 (43.8)	83 (30.3)	2.0 (1.3–3.2)		

*SAM* severe acute malnutrition, *COR* crude odds ratio, *CI* confidence interval, *SD* standard deviation, *AOR* adjusted odds ratio, Statistically significant difference at minimum level of *p* = 0.05

### Household risk factors for SAM

Multivariable analysis on the household-level risk factors showed that the odds of SAM was significantly higher among children whose caretakers were undernourished (MUAC < 220 mm) [AOR (95% CI) = 2.6 (1.2–5.5)], caretakers’ habit of less frequent hand washing after defecation/ using toilet [AOR (95% CI) = 1.9 (1.2–3.1)], absence of toilet in the household [AOR (95% CI) = 1.9 (1.1–3.6)], marriage status of caretakers (not married/lives alone) [AOR (95% CI) = 7.7 (2.0–30.1)], and low household food diversity [AOR (95% CI) = 1.8 (1.0–3.1)] (Table 3). Recent morbidity in the household, early pregnancy, hand washing behaviour score, destination of child’s faeces (outside the house), monthly hygiene expenses < 2000 FCFA, educational status of caretaker (did not attend any school), monthly financial resources < 35.000 FCFA, and food insecurity showed an association with SAM in bivariate analysis, but were not significantly associated in the multivariable analysis.

**Table 3:** Household risk factors for SAM, Mao health district, February–August 2017

Characteristic	Cases (%)	Controls (%)	COR (95%CI)	AOR (95%CI)	*P*-value
Recent morbidity in the household	42 (30.7)	59 (21.5)	1.66 (1.0–2.7)		
MUAC of mother/caretaker < 220 mm	19 (13.9)	14 (5.1)	3.0 (1.4–6.1)	2.6 (1.2–5.5)	0.015
Early pregnancy ≤18 years	29 (21.2)	33 (12.0)	1.9 (1.1–3.2)		
Hand washing behaviour score, mean (SD)	6.8 (1.0)	7.0 (0.9)	0.8 (0.6–1.0)		
Not washing hands after defecation/using toilet	74 (54.0)	110 (40.2)	1.8 (1.2–2.8)	1.9 (1.2–3.1)	0.009
Absence of toilet in the household	78 (56.9)	129 (47.1)	2.3 (1.2–4.1)	1.9 (1.1–3.6)	0.046
Destination of child’s faeces, outside the house	117 (85.4)	216 (78.8)	1.9 (1.0–3.8)		
Monthly hygiene expenses < 2000 FCFA	66 (48.2)	99 (36.1)	2.0 (1.2–3.4)		
Marriage status of caretaker /lives alone	9 (6.6)	3 (1.1)	6.0 (1.6–22.1)	7.7 (2.0–30.1)	0.003
Caretaker did not attend any school	18 (13.1)	18 (6.6)	2.9 (1.2–6.6)		
Monthly financial resources < 35.000 FCFA	75 (54.7)	123 (44.9)	1.5 (0.9–2.3)		
Food insecurity	46 (33.6)	63 (23.0)	1.9 (1.1–3.1)		
Household Food Diversity Score ≤ 6	64 (46.7)	100 (36.5)	1.9 (1.1–3.2)	1.8 (1.0–3.1)	0.041

*SAM* severe acute malnutrition, *COR* crude odds ratio, *CI* confidence interval, *SD* standard deviation, *AOR* adjusted odds ratio, Statistically significant difference at minimum level of *p* = 0.05; *MUAC* mid upper arm circumference, *FCFA* central african franc, 1 XAF = 0,00152 EUR

## Discussion

Our study investigated individual and household risk factors for SAM among children aged 6–59 months in Mao health district, Kanem region, Chad. Using a matched case-control design, we identified and quantified the effect of several risk factors at both levels. The results point to the need for a package of interventions to address infections at the child level through appropriate treatment and by intervening on hygiene and socio-economic status of the household.

We found a strong association between recent morbidity in a child and SAM. This result supports the evidence on the well-accepted link between infections and nutritional status of children, as they affect dietary intake, uptake and utilization. Interactions between infections and undernutrition have long been described as a vicious circle, one being a risk factor for the other, and vice versa [17–19]. These results are in line with the findings from another study conducted in south-eastern Chad [20] which showed that recent episodes of diarrhoea, fever and pneumonia were strongly associated with SAM. This finding points to the importance of treating infections in a prompt manner (for example with Oral Rehydration Salt in case of diarrhoea and vomiting), and preventing infections in children through an integrated approach to tackle environmental health, household socio-economic status and caretaker’s habits.

Absence of toilet in the household and caretakers’ hand washing habits were significantly associated with both SAM and diarrhoea. This is consistent with the existing evidence [21–23]. Poor access to sanitation and failure to wash hands after defecation increase the risk of faecal contamination of home surroundings, which leads to ingestion of faecal pathogens causing infections such as diarrhoea. This ultimately results in increased risk of undernutrition and SAM. Findings from a recent study conducted in Bangladesh are in concordance with these results [24]. Actions aiming at improving WASH conditions and hygiene practices at the household level are therefore a priority.

Similarly, contaminated water increases the risk of direct ingestion of faecal material [25]. To our surprise, we did not observe a significant association between point-of-use water quality and SAM. None of the households included in the study had a good drinking water quality. Probably, cases and controls had access to the same water source, as one of the recruitment criteria was proximity. This universal exposure could have hidden a real risk. Dose-response analysis of increasing levels of contamination also did not show any association. Our findings may indicate that in this context pathogens are rather transmitted through poor sanitation and hand hygiene than through poor quality of drinking water, as reported in Nigeria [26].

The results from this study indicated that the type of complementary meal introduced (family dish) was significantly associated with SAM. “Family dish”, a cereal based porridge with okra and meat sauce, was probably inadequate in terms of energy, protein and micronutrients, or nutrient density in relation to the child’s needs [27]. In the Kanem region, family dish is usually served in large plates, from which all family members eat together by hands while sitting on mats or carpets. Such practice could also represent a transmission pathway of enteric infections through unhygienic feeding practices and poor hand hygiene of caretakers and other family members [28, 29].

In our study, the odds of SAM were significantly higher among children who were stunted. While the causal relation between stunting and wasting is little explored and stunting cannot be clearly identified as a direct risk factor for SAM [30], experiencing chronic nutritional deficits greatly increases susceptibility to and severity of infections, which are proximate determinants of SAM.

Results of the present study also showed that maternal undernutrition increases the risk of having a malnourished child, which is consistent with other research findings [31, 32]. Poor maternal nutrition has a detrimental impact on nutritional status of a child at the foetal stage and beyond. One negative consequence is in-uterus growth retardation, which results in infants being born small for gestational age. Evidence shows a negative association between small for gestational age and future child nutritional status [33, 34]. Unfortunately, data on children’s weight at birth were not available, so we could not explore this association. However, in our context, poor nutritional status of both mother/caretaker and child are likely to be related to the low socio-economic status of the household [35]: risk of SAM was higher for children in households with one bread-winner and with the low household food diversity score. These observations are in line with previous findings [36], which showed that lower food diversity score was associated with significantly higher likelihood of wasting. However, this association was found significant in rural areas but not in urban and further research is needed to confirm and clarify relationship between various food diversity indicators and SAM.

With regard to the caregiver’s marital status and its association with SAM, the very broad confidence interval reflects the paucity of cases we encountered. This result should therefore be considered with caution. While the link between women’s social position and child nutrition is well recognised [37–39], future studies investigating position of women, autonomy and capabilities in the household and in the community could better shed lights on power relations and the mechanisms linking marital status and child undernutrition in this context.

While this is the first study on the individual and household-level risk factors for SAM in the Kanem region, it has some limitations. The study relied on participants’ self-reported data, which is prone to recall bias and tendency of respondents to report socially desirable behaviours. However, as this applies to both groups, the possible bias should not affect the strength of associations. Due attention was given to respecting study procedures, including training of data collectors, taking anthropometric measurements and supervision throughout data collection period to minimize this expected bias. Overmatching might have occurred and hindered the identification of risk factors associated with the matching criteria (proximity). Matching on neighbourhood matches on a large number of “implicit” factors (e.g. altitude, climate, food-economy, ethnicity, socio-economic status, extended family, religion, etc.). This means that the association between SAM and these factors could not be, and was not, explored. It may also mean that risk factors that may also associated with location (e.g. water source) may not be identified. Matching on age and sex, however, expands the “search radius” required to find controls. Having more than one matched control per case has the same effect. These procedures help to reduce problems of overmatching when neighbourhood controls are used.

In this study design, the ascertainment of exposure is done after the outcome. This means results lack the element of temporality and therefore causality cannot be implied. Recruiting cases at the health centre may have introduced a self-selection bias, as cases who sought care at the health centre may be different than cases who did not. SAM symptoms may have alerted caretaker to go to the health centre, overestimating the odds ratio between recent morbidity and SAM. Nevertheless, this was considered a suitable approach to investigate a rare condition such as SAM. In fact, recruiting cases in the community would have been a long and costly process, and recruiting controls in the health centre was not appropriate to our objective of matching on the place of residence (village/ neighbourhood).

Finally, the data is context specific and special consideration must be taken on extrapolating the findings to other contexts.

## Conclusions

The present study identified the need for an integrated approach to address infections at the child level through appropriate treatment and by intervening on hygiene and socio-economic status of the household. It showed that child morbidity, stunting, type of first complementary meal introduced, marriage and nutritional status of caretaker, hygiene practices and absence of toilet in the context without access to safe drinking water, and low household food diversity are the risk factors for SAM in Mao health district, Kanem region, Chad.

Based on these findings, actions should be designed to prevent and treat childhood illnesses, improve child care and feeding practices, ameliorate sanitation conditions in the households and hygiene practices of caretakers, including hygienic food preparation. Programs focusing on improving women’s nutritional status, along with increasing household food diversity are also likely to lead to improved nutritional status of children in this setting.

## Abbreviations

AOR: Adjusted odds ratio
ASRADD: Alliance Sahelienne de recherches appliquées pour le developpement durable
CI: Confidence interval
COR: Crude odds ratio
FCFA: Central african financial cooperation franc
GAM: Global Acute malnutrition
HAZ: height-for-age Z-score
INGO: International non-governmental organization
MUAC: Mid upper arm circumference
NTU: Nephelometric turbidity unit
OR: Odds ratio
OTP: Outpatient therapeutic program
SAM: Severe acute malnutrition
SD: Standard deviation
SHPERE: Minimum standards for the delivery of quality humanitarian response
UNICEF: United nations children’s fund
VIF: Variance inflation factors
WASH: Water, sanitation and hygiene
WHO: World health organization
WHZ: Weight for Height Z-score
